# Symptoms and Diagnosis

**Published:** 1912-05-11

**Authors:** 


### THE MAKING OF THE MODERN PRACTITIONER.

### Symptoms and Diagnosis.

THE treatment of symptoms as such is rightly condemned by those who teach medical students the rudiments of clinical medicine and surgery. And it is the philosophic and scientific spirit of inquiry into prime causes which accounts not only for the immense strides of medical and surgical knowledge in recent decades, but also for the general levelling up of the standard of achievement in the profession at large.

### SYMPTOMS IN PRIVATE PRACTICE.

At the same time the practitioner soon discovers in his practice that the ideals he has been taught to aim at in hospital wards are by no means always fully attainable outside; or, at least, are not always immediately attainable. He finds that, will he nill he, symptoms must be relieved if he is to retain the confidence of his patients, and that to the multitude such a prudent course as the suspension of judgment in an obscure case appears merely to be a sign of ignorance or lack of ability. Experience rapidly teaches him that he must adapt himself to circumstances, and eventually he comes to see that in most cases he can steer a middle course between mere peddling opportunism, with neglect of the real interests of his patients, and a cold-blooded scientific detachment which results in the loss of their affections and of his own revenues.

The importance of symptoms, therefore, to the general practitioner is twofold. He must acquire the most intimate knowledge of their assuagement and also of their interpretation. He can afford to overlook the latest improvement in the technique of, say, gastro-enterostomy; but on no account may he forget the rational treatment of an ache or pain in any region of the body. He must be a mine of clinical "wrinkles," recognising the limitations of the older clinical methods when he reaches them, but able to diagnose a straightforward case of any fairly common disease without the assistance of a whole series of blood determinations and indices. Above all things, in fact, he must retain a clear memory of the possible causes of leading symptoms, pigeon-holed in well classified order and mentally ranged in the scale of their comparative frequency.

Perfection in such a complicated and difficult task is not to be looked for within the compass of a single brain. In text-books the classification is by diseases, not symptoms; so that Dr. French's Index* may be said to open up new ground. It is true that Dr. Leftwich long ago compiled an Index, which is, in skeleton outline, a predecessor of this bulky volume. To that extent the idea is not new, but its treatment under Dr. French's editorship has resulted in something quite unique. If one could imagine the ideal general practitioner replete with practical experience on every subject and every specialty, every symptom and every disease classified in his mind with superhuman accuracy, this book might represent his attempt to put on paper his mental processes on encountering any symptom of disease that man or woman may exhibit.

The book, says the editor, is a treatise on the application of differential diagnosis to all the main signs and symptoms of disease, and it covers the whole field of medicine, surgery, gynæcology, ophthalmology, dermatology, and neurology. It is designed as a companion volume to the "Index of Treatment" edited for the same publishers a few years ago by Hutchison and Collier. Unlike that excellent text-book, this one is the work of quite a small band of contributors; they are chiefly young consultants, but they are also men who have already made a mark as able clinicians and expositors.

The object is to afford assistance to the baffled practitioner who is faced with a case in which the diagnosis of the lesion underlying a symptom or series of symptoms is for the time being obscure. The leading symptom or symptoms having been turned up in its alphabetical place a comprehensive survey is found of the differential diagnosis of the diseases to which the symptom may be due, together with suggestions for lines of investigation with a view to clearing up the case. Treatment is, of course, excluded, for the whole aim and object is the facilitation of exact diagnosis. But lest the discussion of symptoms should mislead the reader into exalting them into the chief place of honour, a general index is provided in which the classification is primarily by diseases, not by symptoms. The importance of this general index in the scheme of the work may be gathered from the fact that it occupies 167 pages of very small type, most compactly arranged, and contains about 45,000 entries.

### MINOR CRITICISMS.

Than Dr. French probably no better editor for the purpose could have been secured. He himself is the most voluminous contributor to the book, and his articles are storehouses of information upon the causation of the symptoms with which he deals. In so very large and comprehensive a work as this it is only to be expected that small sins of omission or commission may seem apparent to the individual critic. To give an example, the cramps so common in simple anæmia and in labour are not mentioned in the article on that symptom. But taking a broad view it may justly be said that the imperfections are amazingly few, and that the excellence of the articles is such as to command the highest encomiums. To the general practitioner Dr. French's Index will, we feel sure, be a very present help in time of trouble, especially to those who feel the proper artistic and professional satisfaction in tracing to their exact origin the symptoms and signs of an obscure and puzzling case of disease. We predict for it an assured and immediate success, for it is full value for the price at which it is published.

* *An Index of Differential Diagnosis of Main Symptoms.* Edited by Herbert French, M.D., F.R.C.P. Bristol: J. Wright and Sons, Ltd. 1912. Pp. 1017. Price 21s. net.

